# A Robust Deep Neural Network for Rolling Element Fault Diagnosis under Various Operating and Noisy Conditions

**DOI:** 10.3390/s22134705

**Published:** 2022-06-22

**Authors:** Chun-Yao Lee, Guang-Lin Zhuo, Truong-An Le

**Affiliations:** 1 Department of Electrical Engineering, Chung Yuan Christian University, Taoyuan 320314, Taiwan; s10528245@cycu.org.tw; 2 Department of Electrical and Electronic Engineering, Thu Dau Mot University, Thu Dau Mot 75000, Binh Duong, Vietnam; anlt@tdmu.edu.vn

**Keywords:** intelligent diagnostic, bearing faults, improved fast kurtogram (IFK), nonlinear mode decomposition (NMD), gramian angular field (GAF), convolutional neural network (CNN)

## Abstract

This study proposes a new intelligent diagnostic method for bearing faults in rotating machinery. The method uses a combination of nonlinear mode decomposition based on the improved fast kurtogram, gramian angular field, and convolutional neural network to detect the bearing state of rotating machinery. The nonlinear mode decomposition based on the improved fast kurtogram inherits the advantages of the original algorithm while improving the computational efficiency and signal-to-noise ratio. The gramian angular field can construct a two-dimensional image without destroying the time relationship of the signal. Therefore, the proposed method can perform fault diagnosis on rotating machinery under complex operating conditions. The proposed method is verified on the Paderborn dataset under heavy noise and multiple operating conditions to evaluate its effectiveness. Experimental results show that the proposed model outperforms wavelet denoising and the traditional adaptive decomposition method. The proposed model achieves over 99.6% accuracy in all four operating conditions provided by this dataset, and 93.8% accuracy in a strong noise environment with a signal-to-noise ratio of −4 dB.

## 1. Introduction

As one of the basic components of modern industry, rotating machinery must ensure the reliability of its operation, among which bearings are an important part to maintain stability [1]. An IEEE study shows that bearing in induction machines fails most frequently, accounting for 42% of the total [2]. The early detection of failures to reduce maintenance costs and prevent unplanned downtime is a top priority for operators [3]. Therefore, it is an urgent task to develop a diagnostic system that can identify fault signals of motor bearing as early as possible.

With the continuous development of Big Data technology, industrial systems collect a large amount of operating data through sensors. How to effectively use these data is a major challenge for diagnostic methods [4]. Therefore, data-driven diagnostic methods have been proven to effectively utilize these signals to achieve accurate fault diagnosis [5]. Numerous studies on fault diagnosis have been published in recent years [6]. Song et al. proposed a signal analysis method combining statistical filter and wavelet packet transform with moving peak hold method, which can effectively extract weak fault signals of low-speed machinery [7]. Special bearing diagnostic symptom parameters are defined to extract sensitive features from the frequency domain. Van et al. combined non-local mean denoising and empirical mode decomposition (EMD) to accurately extract fault features [8]. The two-stage feature selection of hybrid distance evaluation technology (DET) and particle swarm optimization (PSO) can effectively divide the feature interval and find the best feature subset. Wang et al. proposed a model that integrates fault diagnosis and prediction [9]. The model adopts the multi-scale envelope spectrum to analyze the fault characteristics and expresses it as a bearing health index, and then estimates the residual life by Bayesian inference. Despite the success of the above studies, the main limitations of these methods are that the feature extraction process is highly dependent on expert experience and experiments, and the diagnostic accuracy is highly dependent on the quality of the selected features [10]. The process of manually selecting features is usually time-consuming and only suitable for specific tasks [11]. In addition, the fully connected structure of feature extraction, feature selection, and classifier is not deep enough, which is a challenge for the classification task of complex systems [12]. Therefore, deep learning (DL), which can automatically complete feature extraction and has a deep structure capable of learning the complex relationship between signals and features, is the current research trend [13].

Recently, a large number of bearing fault diagnosis studies using DL have been proposed [14]. Among them, the convolutional neural network (CNN) is particularly suitable for developing bearing intelligent diagnosis models due to its sparse connection, weight sharing, and performance in processing periodic signals [15,16]. Huang et al. proposed a multi-scale cascaded CNN (MC-CNN) to find useful frequency bands through filters of different scales to enhance the input information [17]. Li et al. proposed a method for intelligent bearing remaining useful life prediction using a short-time Fourier transform (STFT)-based time–frequency map combined with CNN [18]. Darong et al. proposed to combine a modified recursive least squares (RLS) model based on the momentum factor and the forgotten factor with local mean decomposition (LMD) for early bearing fault diagnosis [19]. Zhao et al. proposed a deep rational attention network (DRANet) for fault diagnosis. In this method, signal denoising is introduced and the proposed pseudo-soft threshold function is used to avoid gradient vanishing [20]. However, the robustness of these studies to noise and variable operating conditions is still insufficient. Because motors often operate at variable speeds and noisy environments in industrial environments, these strong interference components mask the fault pulses, making the traditional CNN-based fault diagnosis models have poor fault diagnosis performance under variable conditions. Tang et al. propose a multiscale CNN that integrates a vision transformer (ViT) and continuous wavelet transform (CWT). The model integrating CWT and ViT can offer more hidden fault-related information from multi-scale components and achieve higher generalization and anti-noise performance [21]. Qiao et al. proposed an adaptive weighted multiscale convolutional neural network (AWMSCNN) to address the domain shift problem that may be caused by fault diagnosis under variable working conditions. The AWMSCNN with several convolution kernels of different widths and adaptive weight vectors has strong fault discrimination ability and domain adaptation ability under variable working conditions [22]. Qin et al. proposed a deep twin convolutional neural networks with multi-domain inputs (DTCNNMI), which integrated time–domain, time–frequency domain, and time–domain statistical features. DTCNNMI was successfully validated under strong noise and different operating conditions [23]. Qiao et al. proposed a dual input of time–domain signals and time–frequency images combined with CNN and long short-term memory (LSTM) to study fault diagnosis under variable loads and different noise conditions [24]. Jin et al. proposed a CNN with an attention mechanism and adopted a random sampling strategy and an exponential linear unit as the activation function to improve the adaptability of the network under complex conditions [25]. Zhang et al. proposed a CNN with wide first-layer kernels to extract features and suppress high-frequency noise using wide kernels in the first convolutional layer, namely WDCNN [26].

Based on the above review, although many diagnostic models can achieve good classification results, not all diagnostic models can achieve high-precision diagnosis in complex environments, especially under noisy and changing load conditions, and there are still relatively few studies in this part. Ensuring classification efficiency in complex environments is the goal of this study. Therefore, this study introduces a mono-component decomposition method with strong noise immunity, called nonlinear mode decomposition (NMD) [27]. NMD integrates time–frequency analysis [28], surrogate data testing [29], and harmonic identification [30]. Research on the fault diagnosis of rotating machinery [31,32] has demonstrated its noise robustness and only extracts physically meaningful oscillations. Moreover, an improved fast kurtogram (IFK) is proposed to address the computational inefficiency of NMD pointed out by [32]. Furthermore, the classification performance of CNN is highly dependent on dataset quality. Therefore, the gramian angular field (GAF) is introduced to construct a 2D image, which can obtain a unique temporal correlation mapping in polar coordinates [33]. In summary, this paper proposes an intelligent fault diagnosis model, called IFKNMD-CNN, which uses CNN to classify a dataset constructed by an innovative combination of IFK, NMD, and GAF. The advantages and contributions of this paper are summarized as follows.

1. The combination of IFK and NMD not only greatly improves the computational efficiency, but also has a high tolerance to noise. Because IFK finds the best frequency band of the signal, it filters out redundant parts and noise in the signal.
2. NMD uses a surrogate data test to ensure that the output is a physically meaningful component rather than noise; therefore, the fault diagnosis model can achieve robust performance even in highly noisy environments.
3. GAF can obtain a unique time map in the polar coordinate system, fully demonstrating the advantages of CNN in classifying bearing signals.
4. This study uses the public dataset provided by Paderborn University to verify the effectiveness of the model [34]. The performance of the proposed model is validated in comparison with other state-of-the-art methods using the same dataset. Furthermore, the proposed model is tested under the four operating conditions provided by the Paderborn dataset, proving that the model also has a high degree of generalization.

The rest of this article is organized as follows. Section 2 presents the theoretical background of the proposed model. Section 3 details the performance improvement of the proposed method and the diagnostic steps of the proposed model. Section 4 discusses the experimental results on the Paderborn University dataset. Section 5 carefully evaluates the model. Finally, Section 6 summarizes the fault diagnosis model.

## 2. Background Theories

### 2.1. Nonlinear Mode Decomposition

The merits of NMD in adaptive mode decomposition and anti-noise capability are based on a powerful combination of time–frequency analysis, surrogate data test, and harmonic identification [27]. Detailed descriptions of the NMD are as follows:

*Step* *1:* The time–frequency representation (TFR) of a given vibration signal *x*(*t*) is calculated from the windowed Fourier transform (WFT) defined as *G_x_*(*ω*,*t*). Next, the dominant component is extracted from the TFR, and its characteristic parameters (including instantaneous amplitude, instantaneous phase, and instantaneous frequency) are reconstructed by the ridge method. The corresponding formulas of the ridge method are as follows:

(1) $$\omega_{p}\left(t\right)=\arg\max_{\omega }\left|G_{x}(\omega ,t)\right|$$

(2) $$\omega \left(t\right)=\omega_{p}\left(t\right)+\delta \omega_{d}\left(t\right)$$

(3) $$A\left(t\right)e^{i\phi \left(t\right)}=\frac{2G_{x}\left(\omega_{p}\left(t\right),t\right)\;}{\hat{g}\left[\omega_{p}\left(t\right)-\omega \left(t\right)\right]\;}$$

(4) xd(t)=Re[A(t)eiϕ(t)] 

where *ω*_*p*_(*t*) is the ridge curve of the dominant component, *δω*_*d*_(*t*) is the correction for discretization effects, the Gaussian window for the WFT is denoted as $\hat{g}(w)=e^{-{\left(f_{0}w\right)}^{2}/2}$, and *x*_*d*_(*t*) is the dominant component to be extracted.

*Step* *2:* The Fourier transform (FT) surrogate test can effectively identify the reference component or noise. This method is constructed by taking the inverse Fourier transform of the signal’s FT and randomizing the phase of the Fourier coefficients:

(5) $$x_{x}\left(t\right)={\left(2\pi \right)}^{-1}\int \left[\hat{x}(\xi )e^{i\phi_{x}(\xi )}\right]e^{i\xi t}d\xi$$

where *ϕ_x_*(ξ) represents a uniformly random phase taken on [0, 2π]. If the reference component is true, then it should be more deterministic, since randomization will destroy the phase relationship of the amplitude and frequency modulation of the surrogate component, making it less deterministic.

Spectral entropy quantifies the degree of determinacy in the extracted amplitude *A*(*t*) and frequency *ω*(*t*), and the combination of the spectral entropies is the discriminant statistic for the surrogate test. The formulas of spectral entropy and discriminant statistics *D* are defined as:

(6) $$Q\left[f(x)\right]\equiv -\int \frac{{\left|f(x)\right|}^{2}}{\int {\left|f(x)\right|}^{2}dx}\log\frac{{\left|f(x)\right|}^{2}}{\int {\left|f(x)\right|}^{2}dx}dx$$

(7) $$D\left(\alpha_{A},\alpha_{\omega }\right)\equiv \alpha_{A}Q\left[\hat{A}\left(\xi \right)\right]+\alpha_{\omega }Q\left[\hat{\omega }\left(\xi \right)\right]$$

The significance *D_s_* is defined as the maximum value among *D*(1,0), *D*(0,1), and *D*(1,1). *D*_0_ is the significance of the original component. In this paper, *N_s_* = 40 FT surrogates are built, and the significance level is set to *λ* = 95%. The corresponding significance *Ds* = 1, 2, …, *N_s_*(*α_A_*,*α_ω_*) is calculated and compared with the original components. If the surrogates for *D_s_* > *D*_0_ are not less than *λN_s_*, the original component is considered true.

*Step* *3:* The time–shift surrogate test is used to check whether the dominant component *x_d_*(*t*) can represent the first harmonic (fundamental). The time-shifted surrogate method can build harmonic surrogates consistent with the null hypothesis of independence and reduce the interference caused by noise, finite frequency, and time resolutions. In the time–shift surrogate test, *x_d_*(*t*) is assumed to be the fundamental harmonic *x*_1_(*t*). Next, the corresponding candidate harmonics for *i* = 1/2, 1/3, … are extracted from the time-shifted TFR. The formula for the instantaneous amplitude *A_i_*(*t*) of the subharmonic *x_i_*(*t*) is constructed by shifting Δ*T_d_*/2 backward:

(8) $$A_{d}^{i}\left(\tau \right)=A^{i}(\tau -\Delta T_{d}/2)$$

(9) $$\tau =t_{i}{}_{=1+M/2,\ldots ,N-M/2}$$

(10) $$\Delta T_{d}{}_{=1,\ldots ,N_{d}}=M\left(1-2d/N_{d}\right)/2f_{s}$$

where *N* is the total length of the subharmonic, *M* is the maximal time–shift, *d* represents the index of candidate harmonics, *N_d_* indicates the number of candidate harmonics, and *f_s_* is the sampling frequency of signal.

A metric value $q_{A}^{i}$∈[0, 1] is designed to quantify the degree of dependence between the first harmonic and the extracted harmonic candidates (0: no consistency, 1: full consistency). A metric value of amplitude $q_{A}^{i}$∈[0, 1] is defined as:

(11) $$q_{A}^{i}\equiv \exp\left[-\frac{\sqrt{\langle {\left[A^{i}(t)\langle A^{1}(t)\rangle -A^{1}(t)\langle A^{i}(t)\rangle \right]}^{2}\rangle }}{\langle A^{1}(t)A^{i}(t)\rangle }\right]$$

The overall metric of interdependence between the harmonics is defined as:

(12) $$\rho^{i}={\left(q_{A}^{i}\right)}^{\alpha_{A}}$$

where *α_A_* is the weights of each metric $q_{A}^{i}$. According to (8)–(11), the consistency of the candidate harmonics $\rho_{{d=1,\ldots ,N}_{d}}^{i}\left(1\right)$ is calculated and compared with the value $\rho_{0}^{i}\left(1\right)$ of the zero time shift Δ*T*_0_ = 0 for consistency comparison. The ratio of $\rho_{{d=1,\ldots ,N}_{d}}^{i}{>\rho }_{0}^{i}$ represents the probability that the *i*th harmonic is a true harmonic. In this paper, the probability is set to be ≥95% and the number of candidate harmonics *N_d_* is set to 100. In addition, to reduce the probability of false positives caused by noise, a threshold *ρ*_min_ = 0.25 is set in the time-shifted surrogate test. The harmonics are considered true only if they pass the surrogate test while being greater than the threshold.

*Step* *4:* The true harmonic with the smallest index *i* found in the previous step is the reference component for extracting high-order harmonics. *Step 3* is repeated and the high-order harmonics are stored as *i* = 2, 3, ….
*Step* *5:* The true harmonics are subtracted from the given signal *x*(*t*) and all the above steps are repeated until the stopping condition is met, i.e., the residual is identified as noise.

NMD is a powerful combination based on time–frequency analysis, surrogate data testing, and harmonic identification. NMD extracts dominant components through time–frequency analysis, FT surrogate data tests, and time–shift surrogate data tests, removing interfering components such as noise. A metric is then defined to identify physically meaningful modes. Therefore, NMD achieves adaptive decomposition and robustness to noise. In this study, the instantaneous amplitude in the mode can be extracted and used to construct images further that clearly reflect the fault features of the test motor.

### 2.2. Gramian Angular Field

**Figure 1:**
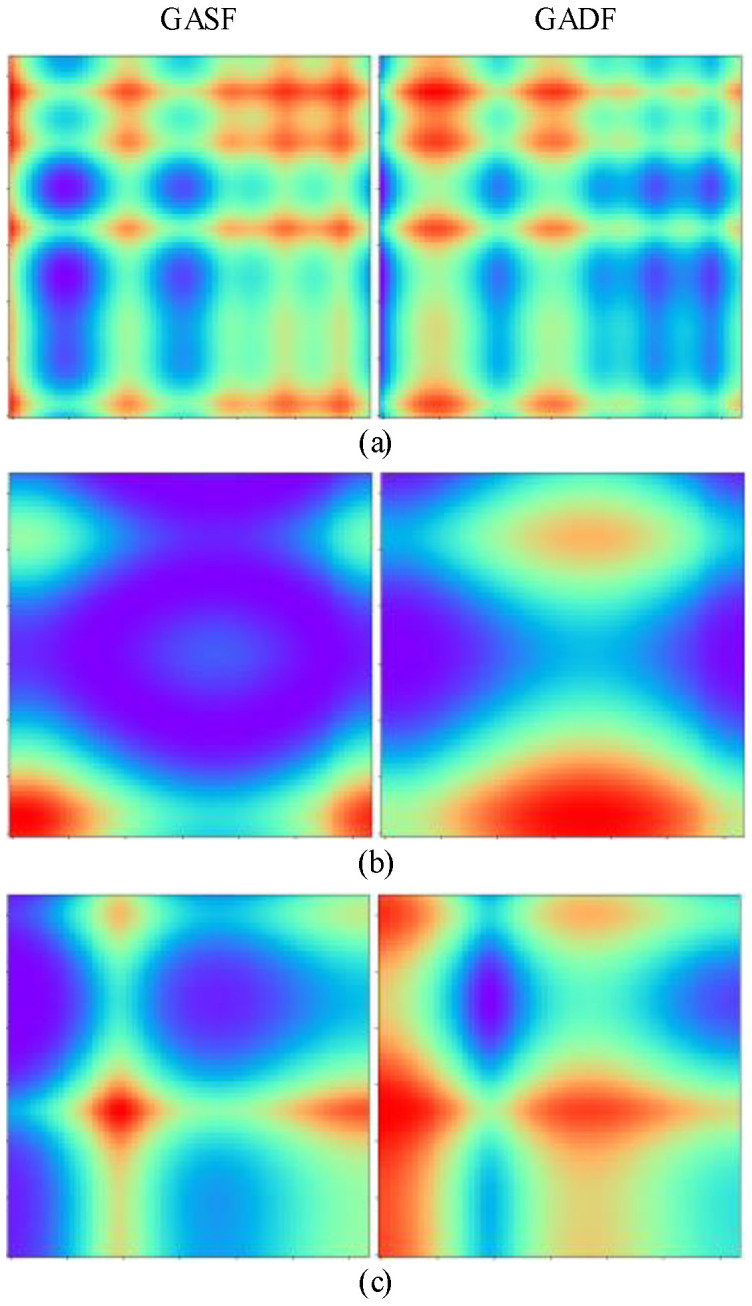
Illustration of GAF image: (**a**) healthy; (**b**) outer ring fault; and (**c**) inner ring fault.

GAF is used to map a time series signal to a polar coordinate system, encoding the signal into a unique time map to help CNNs perform high-precision classification [10]. The time series *X* = {*x*_1_, *x*_2_, …, *x_n_*} with *n* samples is normalized so that all values fall within the interval [−1, 1], as in (13). The normalized time series $\tilde{X}$ is concerted into a polar coordinate system map, with values encoded as the angular cosine (bounded by [0, *π*]) and timestamps as the radius. Note that a given time series produces one and only one map in the polar coordinates system, with a unique inverse map, as in (14). Finally, the trigonometric sum/difference between each sample is calculated to construct an image that preserves the absolute temporal relationship, as in (15) and (16).

(13) $${\tilde{x}}_{i}=\frac{\left(x_{i}-\max(X))+(x_{i}-\min(X)\right)}{\max(X)-\min(X)}$$

(14) $$\{\begin{array}{ll} \phi_{i}=\cos^{-1}({\tilde{x}}_{i}), & -1\leq {\tilde{x}}_{i}\leq 1,{\tilde{x}}_{i}\in \tilde{X}\\ \gamma =t_{i}/N, & t_{i}\in N \end{array}$$

(15) $$\mathrm{GASF}=\left[\cos(\phi_{i}+\phi_{j})\right]$$

(16) $$\mathrm{GADF}=\left[\sin(\phi_{i}-\phi_{j})\right]$$

where *i* is the length of the given signal, *t_i_* is the timestamp, and *N* is a constant used to regularize the span of the polar coordinate system. GAF has the following several advantages: (1) according to (15) and (16), the temporal correlation is represented by superposition/difference for time intervals; (2) in the GAF matrix, the diagonal of the value contains the original value and angular information. Based on the above advantages, GAF can provide high-quality images for CNN to learn the complex relationship between the various health states of the motor. Figure 1 shows the transformation of time series signals into GAF images. Vibration signals from the same bearing dataset are used as experimental results in Section 4. Important features in the signal are highlighted (red part). Moreover, it can be observed from Figure 1 that the important feature distributions of the three states are significantly different, indicating that GAF can clearly express the complex information of rotating machinery.

### 2.3. Convolutional Neural Network

A typical CNN contains numerous modules consisting of convolutional and pooling layers, followed by a fully connected layer. The CNN structure used in this paper contains three convolutional pooling modules, a fully connected layer, and an output layer. The detailed description of the convolution pooling module and the fully connected layer is as follows.

(1) *Convolutional Pooling Module:* In the convolutional layer, a set of convolution kernels consisting of weights and biases performs convolution operations on the input image with a specific stride. Because the same kernel is used to extract features during the convolution operation, the number of neural network parameters can be greatly reduced, and the operation efficiency can be improved. This advantage is called weight sharing. The feature maps are obtained after the convolution operation changes with the weights of the convolution kernel. The activation function is used to perform a non-linear transformation on the feature maps. This operation can help improve model performance. It can be assumed that the convolutional layer *l* has *D* convolution kernels, and *n* = 1, 2, …, *D*. The output of the *n*th convolution kernel $c_{n}^{l}$ can be expressed as:

(17) $$c_{n}^{l}=\mathrm{ReLU}(\sum_{j}I_{j}^{l-1}⊙w_{n}^{l}+b_{n}^{l})$$

where $I_{j}^{l-1}$ is the *j*th output of the previous layer *l* − 1; $w_{n}^{l}$ and $b_{n}^{l}$ are the weights and bias of the *n*th convolutional kernel in convolutional layer *l*; ⊙ indicates the convolution operation; and ReLU represents the activation function.

The pooling layer is usually set after the convolutional layer. The purpose is to down-sample the output of the convolutional layer. The advantage of this operation is to reduce the feature dimension of the output without losing features, which can further improve training efficiency and reduce memory usage. The definition of the max-pooling operation adopted in this study is as follows:

(18) $$P_{n}^{l+1}=\max_{x\times y}(c_{n}^{l})$$

where $P_{n}^{l+1}$ is the output of the max-pooling operation; *x* and *y* represent the size of the pooling region; and the size of the pooling region is set to 2 × 2 in this study.

(2) *Fully Connected Layer:* The fully connected layer can integrate the local features of the motor state obtained in the convolution pooling module and pass it to the output layer for classification. In the fully connected layer, the learned features are flattened into a one-dimensional feature vector, and the classification results are obtained by adjusting the weights and biases. To improve CNN performance, each neuron in the fully connected layer uses an activation function.

## 3. Proposed Method

### 3.1. Speed Acceleration for the NMD Based on IFK

Although NMD is a powerful adaptive decomposition algorithm, the length and frequency range of the signal can seriously affect the computational efficiency of NMD. Therefore, this paper proposes an improved FK (IFK) to find the optimal filtering band of the signal and greatly improve the computational efficiency of NMD. A detailed description of the traditional FK can be found at [35]. In addition, the NMD is limited to searching for candidate components in the optimal filtering frequency band. The IFK greatly improves the diagnostic accuracy using the comprehensive index of the clearance factor and kurtosis. The IFK has three advantages: (1) it resolves any problems after the traditional FK method incorrectly selects the frequency band due to the influence of any other pulse signals in the environment; (2) for rotating machinery, the clearance factor can better separate healthy bearings from faulty bearings; (3) the IFK can extract the fault signal covered by noise and present it as an envelope signal, which can further improve the signal-to-noise ratio (SNR) from the motor vibration signal. The detailed description of IFK is shown in Algorithm 1.
**Algorithm 1:** Improved FK.**Input:** the vibration signal *x*
**Output:** the complex envelope *x_ce_* positioned on the central frequency *f*
1: predefine 1/3 binary tree filter banks; low-pass and high-pass analysis filters *h*_0_(*n)* and *h*_1_(*n*)
2: **for**
*k* = 0 to *L*-1 **do**
3:     define $c_{k}^{i}\left(n\right)$ as the sequence of coefficients obtained from the ith filter // where *i* = 0, …, 2^*k*^ − 1
4:          **if**
*k* = 0: *c*_0_(*n*) ≡ *x*(*n*)
5:          calculate two new coefficients
$c_{k+1}^{2i}\left(n\right)$ and $c_{k+1}^{2i+1}\left(n\right)$ by *h*_0_(*n)* and *h*_1_(*n*)
6:     calculate the kurtosis $K_{k}^{i}$ and clearance factor $C_{k}^{i}$ of all coefficients // where *i* = 0, …, 2^*k*^ − 1
7: **end for *k***
8: calculate the comprehensive index of each coefficient $Q_{k}^{i}$

9: obtain the *x*_*ce*_ based on the coefficient which has the best *Q*


The *h*_0_(*n*) and *h*_1_(*n*) can be represented as $h_{0}{(n)=h(n)e}^{j\pi n/4}$ and $h_{1}{(n)=h(n)e}^{j3\pi n/4}$, respectively; the $Q_{k}^{i}$ is the mean of the statistic values of $K_{k}^{i}$ and $C_{k}^{i}$.

**Figure 2:**
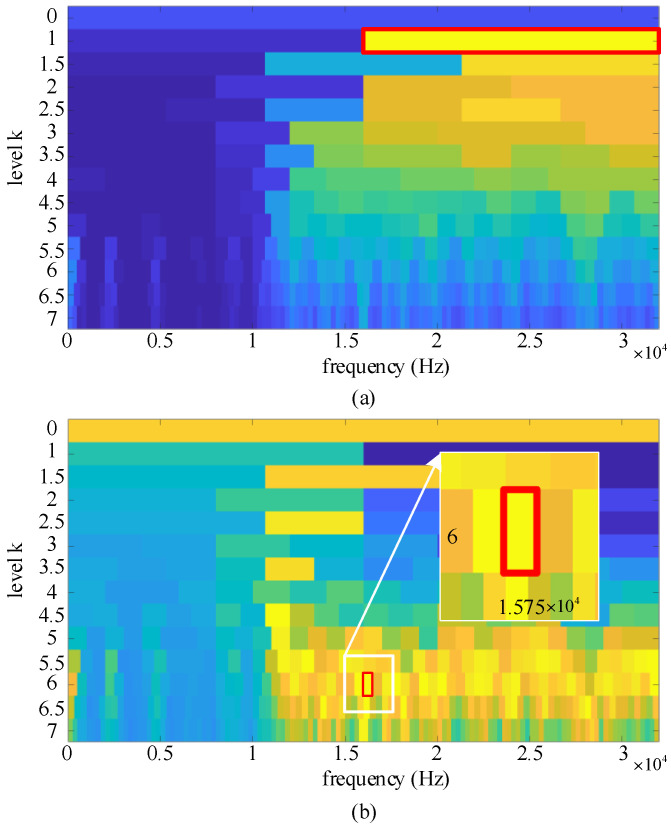
The best frequency band selected by (**a**) FK and (**b**) IFK.

A comparison study of the improved computational efficiency of the NMD method and the original version is carried out. The vibration signal from the same bearing dataset is used as the experimental results in Section 4, and the sampling rate of the signal is 64 kHz. In total, 4 seconds of vibration signal is measured, so the signal length reaches 256,000 data points. In this dataset, bearing codes K, KA, and KI denote healthy bearing, outer ring fault, and inner ring fault, respectively. NMD is limited to searching for candidate components in the optimal filtering band indicated by the IFK. Figure 2 shows the results of selecting the optimal frequency band for the vibration signal of a bearing outer ring fault (KA30). The results show that IFK finds a narrower frequency band. In contrast, FK cannot accurately separate fault pulses or interfering pulses in the environment. In addition, the computational efficiency of the original FK is also compared. The comparison results are shown in Table 1. The original version of the NMD method requires a large amount of time for mono-component decomposition. However, the mono-component extraction for the three bearing states can be accomplished within 22 seconds by the improved NMD method. Moreover, the comparison results with the original FK show that the IFK can find a narrower filtering band to achieve computational efficiency improvements and make it suitable for real-world cases. This comparison study is conducted with an Intel(R) Core (TM) i5-10500 3.1 GHz CPU and 16.0 GB RAM.

### 3.2. Proposed Rolling Element Fault Diagnosis Model

**Figure 3:**
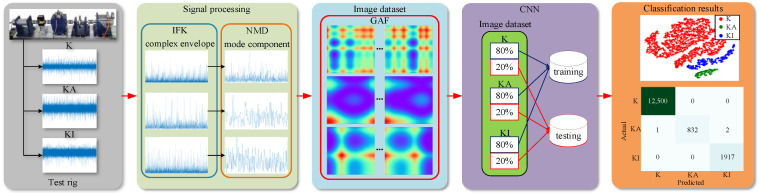
Illustration of a fault diagnosis model.

The proposed induction motor rolling element fault diagnosis model, namely IFKNMD-CNN, is shown in Figure 3. In this model, the vibration signal is obtained from the test rig. Then, the IFK is used to find the best filtering frequency band from the vibration signal and extract the envelope signal. Next, NMD extracts physically meaningful components from the envelope signal and uses GAF to perform 2-D image transformation on the components afterward. Finally, CNN is used for rolling element fault classification. The detailed process of IFKNMD-CNN is summarized as follows.

*Step 1*: The accelerometer is used to collect three kinds of vibration signals from the test platform, including the health, inner ring fault, and outer ring fault.
*Step 2*: The IFK is proposed to find the narrow frequency band containing the main fault information in the vibration signal, filter out the part outside the narrow frequency band through low-pass and high-pass filters, and present the reserved part as an envelope signal, which can greatly reduce the signal length and improve the signal SNR.
*Step 3*: The NMD method is used to further analyze the envelope signal and extract the mode component containing fault characteristics. NMD is constrained to search for components within the optimal frequency band indicated by the IFK. The combination of the IFK and NMD greatly increases computational efficiency.
*Step 4*: GAF is used to transform the time domain signal into a polar coordinate system map and preserve the temporal correlation so that it can construct a high-quality 2D image dataset.
*Step 5*: CNN is adopted to learn the image dataset and perform fault classification. During the classification process, 80% of the samples for each health state of the motor in the image dataset are randomly selected as the training dataset and 20% as the test dataset. Randomly selecting samples can ensure that each classification process uses a different dataset, avoid possible overfitting, and prove the effectiveness of the fault diagnosis model.

## 4. Case Study

### 4.1. Experimental Setup

This study uses the Paderborn dataset proposed by Lessmeier et al. This dataset contains both healthy bearings and widely distributed damage of inner and outer ring bearings. Real damaged bearings are produced by an accelerated lifetime test rig. The damage produced by the lifetime test mainly occurs in different degrees of pitting. Indentations are found on a small number of bearings. A detailed description of the real damaged bearing can be found in [34]. Then, the test bearings are mounted to the modular test rig to generate experimental data. The test rig uses a piezoelectric accelerometer (Model No. 336C04, PCB Piezotronics, Inc., Depew, NY, USA) with a sampling rate of 64 kHz to measure the vibration signal at the adapter at the top end of the rolling bearing module. Each measurement has a duration of 4 seconds and is repeated 20 times independently. The detailed parameter settings of the Paderborn dataset used in this study are shown in Table 2. In this study, data with a length of 128,000 are selected from each fault category for signal analysis (the size of the 2-D image is set to 64 × 64).

### 4.2. Image Dataset for DL Method

Four DL models are compared in this case study, including IFKNMD-CNN, CNN based on LMD with TFR (LMD-TFR-CNN), IFKNMD-1DCNN, and 1D-CNN. In LMD-TFR-CNN, LMD can adaptively decompose the signal into a set of product functions (PFs), and then use PF selection [36] to select the best PF and express it as a time–frequency relationship. IFKNMD-1DCNN is an ablation version of the proposed model, which skips GAF and directly uses one-dimensional signal as the input of CNN. In addition to IFKNMD-CNN and IFKNMD-1DCNN, the data used by both LMD-TFR-CNN and 1D-CNN introduce a wavelet denoising technique to verify the anti-noise capability of the proposed model. In this technique, the selected signal is decomposed using a multi-level decomposition parameterized as a wavelet function (db4) and a hard threshold, and then the signal is reconstructed using an inverse wavelet transform. In this subsection, an operating state, namely the N15_M07_F10 dataset, is used as an example to demonstrate the experimental results.

(1) *Image Dataset for IFKNMD-CNN:* As described in the previous section, the IFK is used to find the optimal frequency band of the signal and represent it as an envelope signal. Table 3 shows the analytical results of the IFK for the bearing categories used in this experiment. The health state (K) has a lower decomposition level, and the faulty state (KA and KI) has a higher decomposition level. This result shows that the IFK can accurately capture the frequency band where the main fault pulses are concentrated, only maintaining the important part of the signal for fault diagnosis. Moreover, the length of the original signal used in this case study is 128,000 and the average length of the extracted envelope signal (15 categories) is 14,712. More than 88% of the unimportant parts of the signal are removed. As verified by the comparison experiments in Section 3, the computational burden of the model in the signal analysis is greatly reduced.

The instantaneous amplitudes obtained from NMD are divided into segments of length 6400. As shown in Table 3, segments with a length of 6400 are kept, and segments less than 6400 are discarded, such as all health state data, KI18, and KI21. If the signal length is less than 6400, it will not be divided, including all outer ring fault data, KI04, KI14, and KI16. Then, each segment is transformed into an image using GAF. If the segment length exceeds 3200, the transformed image size is 3200 × 3200. An image of this size is sufficient to reflect the motor state information without distortion. For all outer ring fault data, KI04, and KI14, the transformed image size is the signal length × signal length. To construct an image dataset of sufficient size to train the CNN, each image is divided into 64 × 64 size images. As shown in Table 4, the dataset contains 76,248 samples. Healthy samples accounted for 81.9% of the total samples. Highly imbalanced datasets are also more suitable for actual applications.

(2) *Image Dataset forLMD-TFR-CNN:* For a fair comparison, the selected best PF is also divided into segments of length 6400, resulting in a total of 20 segments. Each segment is transformed into a TFR (297 × 6400) according to a continuous wavelet transform-based time–frequency analysis. Similarly, each time–frequency image is divided into small 64 × 64 images. Therefore, the dataset has a total of 120,000 samples.
(3) *Image Dataset for IFKNMD-1DCNN:* The instantaneous amplitude obtained from the NMD is divided into segments of length 64. As shown in Table 3, segments whose length is less than 64 are discarded. Therefore, there are 2829 healthy samples, 138 bearing outer ring failure samples, and 475 inner ring failure samples, for a total of 3442 samples.
(4) *Image Dataset for 1D-CNN:* The new signal after wavelet denoising is divided into segments of length 1024, and a total of 125 segments are obtained. There are 15 categories in the bearing dataset, so the dataset has a total of 1875 samples.

### 4.3. Parameters Setting of CNN

**Figure 4:**
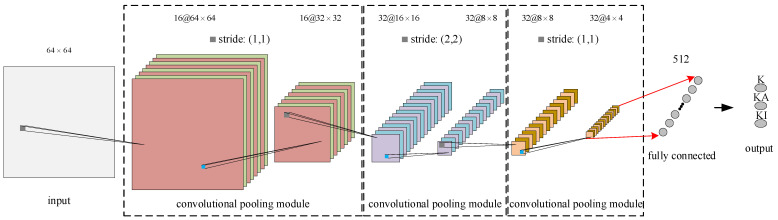
Structure of CNN network.

The images obtained by IFKNMD-CNN (IFK, NMD, and GAF) can clearly reflect the fault features, and then CNN is used to complete high-accuracy fault diagnosis. The structure of the CNN network is shown in Figure 4. The input image size is 64 × 64. The convolution kernels of the three convolutional layers have the same size (3 × 3) and the numbers are 16, 32, and 32, respectively. Padding is set to (1, 1). The activation function adopts ReLU. The number of neurons in the fully connected layer is 512. The output size is 3, corresponding to the number of categories for this case study. The number of training epochs is 500. The Adam algorithm is adopted in the training process with a learning rate of 0.001. This experiment is performed on a computer with Intel(R) Core(TM) i5-10500 3.1 GHz CPU and GEFORCE GTX 1050 GPU running PyTorch 1.10.0.

### 4.4. Intelligent Diagnosis with IFKNMD-CNN

(1) *Performance Analysis of IFKNMD-CNN:* T-SNE and confusion matrix are used in this case study to validate the performance of the proposed model. *Accuracy* and *F*-score are metrics for evaluating model performance, as defined by the following equations:

(19) $$accuracy=\frac{1}{n}\sum_{i=1}^{n}\frac{{\mathrm{TP}}_{i}+{\mathrm{TN}}_{i}}{{\mathrm{TP}}_{i}+{\mathrm{TN}}_{i}+{\mathrm{FP}}_{i}+{\mathrm{FN}}_{i}}$$

(20) $$precision=\frac{1}{n}\sum_{i=1}^{n}\frac{{\mathrm{TP}}_{i}}{{\mathrm{TP}}_{i}+{\mathrm{FP}}_{i}}$$

(21) $$recall=\frac{1}{n}\sum_{i=1}^{n}\frac{{\mathrm{TP}}_{i}}{{\mathrm{TP}}_{i}+{\mathrm{FN}}_{i}}$$

(22) $$F=\frac{2\times precision\times recall}{precision+recall}$$

where *n* represents the three bearing states, and TP, FP, TN, and FN represent true positive, false positive, true negative, and false negative, respectively.

**Figure 5:**
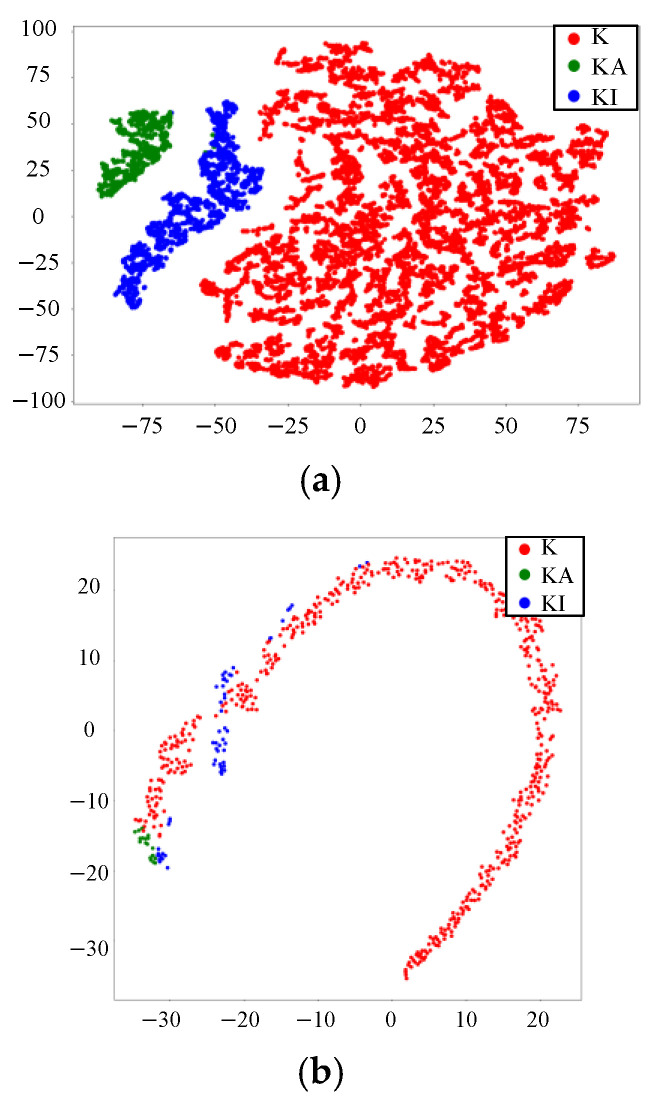
Visualization results with (**a**) IFKNMD-CNN and (**b**) IFKNMD-1DCNN.

**Figure 6:**
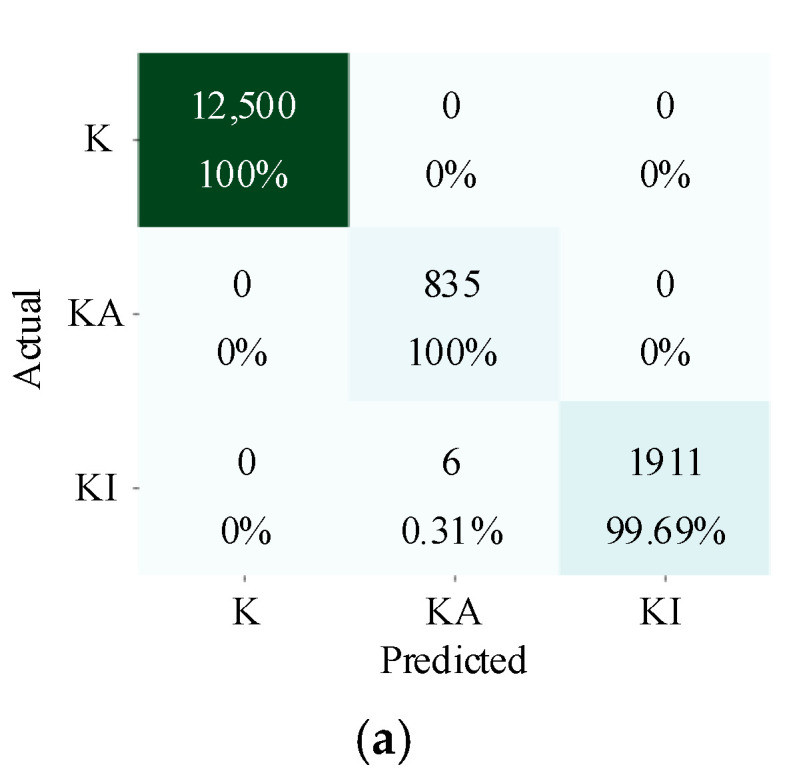

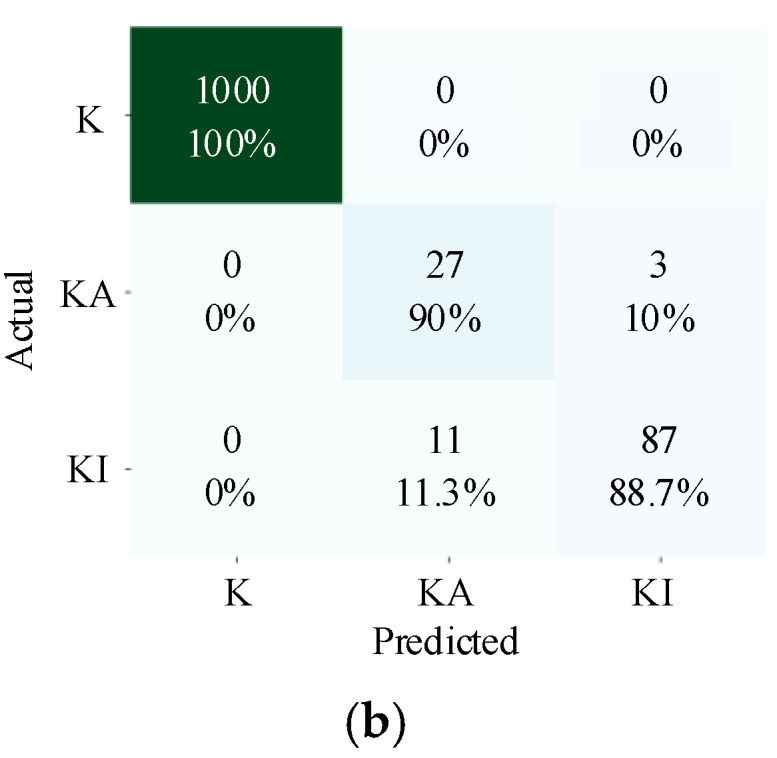
Confusion matrix with (**a**) IFKNMD-CNN and (**b**) IFKNMD-1DCNN.

**Figure 7:**
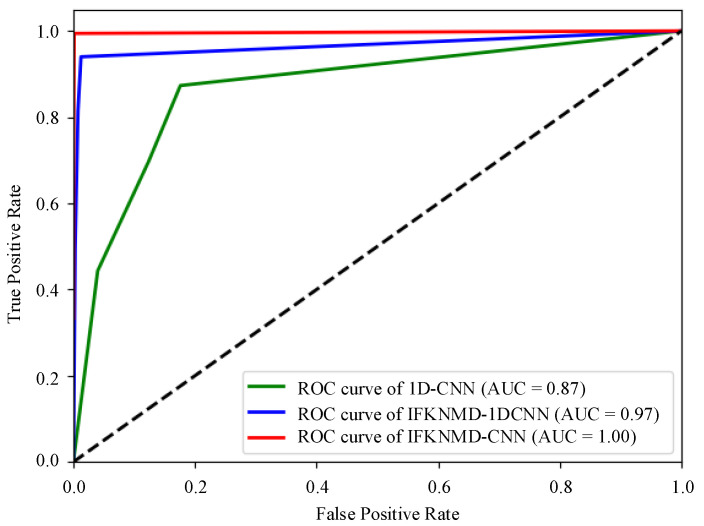
ROC curve with comparison method.

The visualization results of the features of the fully connected layer in the CNN are shown in Figure 5. The features of IFKNMD-CNN (see Figure 5a) are clearly separated. This shows that IFKNMD-CNN can extract useful features to help CNN perform high-accuracy fault classification. In contrast, IFKNMD-1DCNN can distinguish most of the healthy features but cannot effectively distinguish the fault states (KA and KI) (see Figure 5b). The classification results can be more clearly observed through the confusion matrix in Figure 6. Both IFKNMD-CNN and IFKNMD-1DCNN achieve 100% accuracy in recognizing the health state. This indicates that the signals processed by IFK and NMD can clearly distinguish the healthy state. IFKNMD-CNN can separate inner ring fault and outer ring fault more effectively than IFKNMD-1DCNN. In this case study, the accuracy of IFKNMD-CNN (99.96%) is higher than that of IFKNMD-1DCNN (98.41%). This shows that the two-dimensional image obtained by GAF can provide more useful information than the one-dimensional signal. In addition, Figure 7 shows the receiver operating characteristic (ROC) curve as well as the area under the curve (AUC) of the DL method. IFKNMD-CNN achieves the highest accuracy (AUC = 1.00).

In addition, the robustness of IFKNMD-CNN to noise is also evaluated. In this experiment, white Gaussian noise (23) with SNR ranging from −4 to 10 dB is injected into the original signal to construct a new signal corresponding to the SNR.

(23) $${\mathrm{SNR}}_{\mathrm{dB}}=10\log10\left(P_{\mathrm{signal}}/P_{\mathrm{noise}}\right)$$

**Figure 8:**
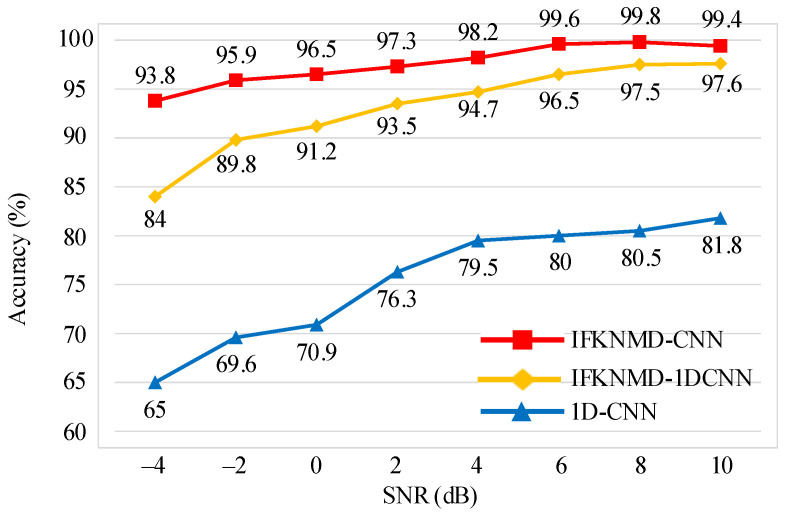
Noise test results with IFKNMD-CNN and comparison methods.

As shown in Figure 8, IFKNMD-CNN and IFKNMD-1DCNN significantly outperform 1D-CNN, while IFKNMD-CNN achieves the best anti-noise capability. The results show that the combination of the optimal band filtering strategy of IFK and the mono-component decomposition of NMD has better anti-noise capability. IFKNMD-CNN achieves an average accuracy greater than 93%. Under the SNR of −4 dB, the accuracy of IFKNMD-1DCNN drops significantly, reaching 5.8%. The overall accuracy of 1D-CNN is lower than that of IFKNMD-CNN, and the fluctuation is obvious. When the SNR value is −4 dB, the accuracy difference between IFKNMD-CNN and 1D-CNN is the largest, reaching 28.8%. There are two factors that lead to these experimental results: (1) the feature similarity between bearing faults (KA and KI) is high, which increases the difficulty of classification; (2) the energy of bearing fault features is very weak, which is difficult to classify correctly in a strong noise environment.

(2) *Comparison With DL Methods:*Table 5 shows the average accuracy and mean *F*-score for 10 independent runs of the four methods. IFKNMD-CNN can identify the health states with almost 100% accuracy. IFKNMD-1DCNN also performs well in identifying healthy states but suffers many classification errors in classifying faulty states (KA and KI). LMD-TFR-CNN introduces a PF function selection technique to remove unimportant PF functions [36]. However, it can be observed from the classification results that LMD is not sensitive to complex relationships between states, and cannot generate high-quality images for CNN to learn features. In addition, although LMD-TFR-CNN and 1D-CNN introduce wavelet denoising to improve the SNR, the wavelet transform is limited by the defined frequency band, which makes it non-adaptive. Although IFKNMD-1DCNN achieves accuracy of 98.62%, the *F*-score is only 87.37%. The main reason for this result is the high similarity between the features of bearing faults (KA and KI), which makes the model unable to classify effectively. The same results can also be observed in 1D-CNN. IFKNMD-CNN successfully highlights the important features of each state by mapping the signal to the polar coordinate system through GAF. In conclusion, IFKNMD-CNN achieves the highest accuracy and *F*-score, proving that the proposed model can achieve robust fault diagnosis.
(3) *Apply to Various Operating Conditions:* This experiment validates the generalization ability of the fault diagnosis model by providing a variety of operating conditions through the bearing dataset. The test rig adjusts rotational speed, load torque, and radial force to construct data for four operating states. The experimental results are shown in Table 6. The accuracies in the four operating conditions are all >99.5%. Therefore, IFKNMD-CNN can be applied to a variety of operating conditions.

### 4.5. Comparison with Existing Methods

The authors of the Paderborn dataset proposed a fault diagnosis method that combines feature extraction, feature selection, and classifiers [34]. Time–domain features, frequency domain features, and time–frequency domain features are first extracted. Then, the maximum separation distance is applied to feature selection. Finally, different classifiers are used for fault classification. In this subsection, three classifiers with the highest classification accuracy are picked out for comparison with IFKNMD-CNN. Furthermore, state-of-the-art fault diagnosis methods based on DL architectures are also compared in this subsection. J Cao et al. proposed a neural architecture search network (NAS), which improves computational efficiency through early stopping and inserts a replay buffer (IRB), and the Pareto efficiency reward function is used to optimize the accuracy, named NAS-PERIRB [37]. L Hou et al. proposed an input feature map (IFM) combined with the residual network (ResNet). The IFM method can extract features without preset parameters [38]. D Wang et al. proposed an attention-based multi-dimensional concatenated convolutional neural network (AMDC-CNN). Important features can be highlighted through the attention mechanism. Multi-dimensional concatenated vibration and torque signals can complement fault features to achieve higher classification accuracy [12].

As shown in Table 7, the accuracy of IFKNMD-CNN is 1.52% higher than the method in [34]. Compared with state-of-the-art DL models, IFKNMD-CNN achieves slightly higher accuracy than NAS-PERIRB and achieves similar performance with IFM-based ResNet and AMDC-CNN. Unlike the above state-of-the-art DL models, this study focuses on proposing an efficient 2D image dataset, so the proposed model does not use the improved CNN architecture for fault classification. The high-quality image dataset used by the proposed model is based on IFK and NMD to extract the most important parts of the signal, and GAF produces high-quality images that preserve absolute temporal relationships. In conclusion, the above proves that IFKNMD-CNN is effective on the Paderborn dataset.

## 5. Discussion

Based on the analysis results of Section 3 and Section 4, the main advantages of this study are as follows. (1) The IFK can select a narrow frequency band that mainly concentrates fault information and can retain the necessary signal part, thus greatly improving the computational efficiency. As shown in Table 1, the time for NMD to complete the mono-component decomposition significantly reduced from 8691 s to 12 s. Moreover, the IFK has more obvious advantages in analyzing the signals of fault types (KA and KI). As shown in Table 4, the IFK selects a decomposition level of at least 3.5 or above, resulting in a reduction in the signal length of more than 91%. The classification results in Section 4 demonstrate that IFKNMD-CNN achieves high-accuracy fault diagnosis despite imbalanced healthy and faulty samples. (2) The advantage of IFKNMD-CNN’s anti-noise capability is based on the IFK and the surrogate data test in NMD. Interfering components such as harmonics and environmental noise in the original signal are first filtered out by the IFK. Then, the carefully designed FT surrogate data and the time–shift surrogate data test the significance and consistency of the signal, respectively. Surrogates that fail the test are rejected, so the noise interference is reduced further.

In addition to the above advantages, IFKNMD-CNN has its limitations. IFK selects the best frequency band by calculating statistical features, and its selection process depends on professional knowledge and the quality of extracted features. Therefore, the performance of the model applied to other datasets may not be as expected.

## 6. Conclusions

In this paper, a novel model is proposed for intelligent bearing fault diagnosis in rotating machinery. The main contribution of this model is to construct an effective image dataset through the combination of IFK-based NMD and GAF. The proposed model uses the IFK to achieve high computational efficiency and improve SNR. A physically meaningful component of the signal is extracted by NMD. Next, the GAF provides images that preserve the absolute temporal relationship of the signal for CNN to perform fault classification. The Paderborn bearing dataset is used to validate the effectiveness of the model. The validation results show that the proposed model achieves more accurate results as well as robustness to noise when compared with LMD methods based on wavelet denoising and PF selection. IFKNMD-CNN achieves competitive accuracy compared to three state-of-the-art DL methods using the same dataset. Furthermore, the proposed model also demonstrates the generalization ability under different operating conditions. Therefore, IFKNMD-CNN has high potential to help rotating machinery apply intelligent diagnosis under strong noise and different operating conditions. However, this study has not been evaluated under variable speed conditions, and the issue of speed domain adaptability remains to be resolved. Transfer learning has strong potential to learn domain-invariant features under variable speed conditions. Therefore, research on transfer learning is an important direction in the future.

## Figures and Tables

**Table 1:** Comparison of computational time.

Bearing Code	Original Signal	FK			IFK		
	t_c_ (s)	Level	f_c_ (Hz)	t_c_ (s)	Level	f_c_ (Hz)	t_c_ (s)
K004	3125	1	24,000	543	4.5	19,333	**22**
KA22	8691	1	24,000	879	6	18,250	**12**
KI14	7322	3.5	30,666	43	6.5	30,167	**10**

The f_c_ and t_c_ indicate central frequency and computational time, respectively. At each level, the signal length of the filtered sequence is reduced by a factor of 2. Therefore, the length of the sequence obtained at level 1 is 127,985, the length of the sequence obtained at level 2 is 63,984, and so on.

**Table 2:** Parameter setting of the experimental dataset.

Rotational Speed [rpm]	Load Torque [Nm]	Radial Force [N]	Name of Dataset
1500	0.7	1000	N15_M07_F10
900	0.7	1000	N09_M07_F10
1500	0.1	1000	N15_M01_F10
1500	0.7	400	N15_M07_F04

**Table 3:** Analysis results of IFK.

Bearing Code	Level	f_c_ (Hz)	Signal Length
K001	1.5	16,000	42,642
K002	3	26,000	15,984
K003	1	24,000	63,985
K004	1.5	16,000	42,642
K005	3	30,000	15,985
KA04	6	30,750	1985
KA15	7	17,375	985
KA16	6	9250	1984
KA22	6.5	16,167	1309
KA30	5.5	24,333	2642
KI04	5.5	14,333	2642
KI14	6.5	24,500	1309
KI16	4.5	18,000	5309
KI18	3.5	28,000	10,642
KI21	3.5	12,000	10,642

**Table 4:** Details of N15_M07_F10 image dataset for IFKNMD-CNN.

Bearing Code	No. Sample	Total Samples	Percent
K001	15,000	62,500	81.9%
K002	5000		
K003	22,500		
K004	15,000		
K005	5000		
KA04	961	4167	5.5%
KA15	225		
KA16	900		
KA22	400		
KA30	1681		
KI04	1681	9581	12.6%
KI14	400		
KI16	2500		
KI18	2500		
KI21	2500		

**Table 5:** Comparison results with deep learning methods.

Method	*Accuracy* (%)				*F* (%)
	K	KA	KI	Overall	
IFKNMD-CNN	**100**	**98.3**	**99.35**	**99.82**	**99.31**
IFKNMD-1DCNN	99.95	91.58	76.11	98.62	87.37
LMD-TFR-CNN	88.58	97.27	93.67	93.17	93.38
1D-CNN	97.2	85.68	78.64	87.17	87.14

**Table 6:** Experimental results in different operating conditions.

Dataset	*Accuracy* (%)				*F* (%)
	K	KA	KI	Overall	
N15_M07_F10	100	98.3	99.35	99.82	99.31
N09_M07_F10	100	98.63	97.15	99.65	98.55
N15_M01_F10	100	98.38	96.74	99.6	98.41
N15_M07_F04	100	96.21	97.43	99.67	97.91

**Table 7:** Accuracy of existing methods.

Method		*Accuracy* (%)
Machine learning method in [34]	CART	98.3
	RF	98.3
	Ensemble	98.3
NAS-PERIRB [37]		99.43
IFM-based ResNet [38]		99.7
AMDC-CNN [12]		99.8
The proposed model		**99.82**

## Data Availability

Not applicable.
